# Supplementary material for: Mapping and Characterization of QTLs for Awn Morphology Using Crosses between “Double-Awn” Wheat 4045 and Awnless Wheat Zhiluowumai
Source: Plants (Basel). 2021 Nov 26;10(12):2588. doi: 10.3390/plants10122588 (PMC8703630; doi:10.3390/plants10122588)
Supplement: Supplementary file 1 [file plants-10-02588-s001.zip › plants-1459628-supplementary.pdf]

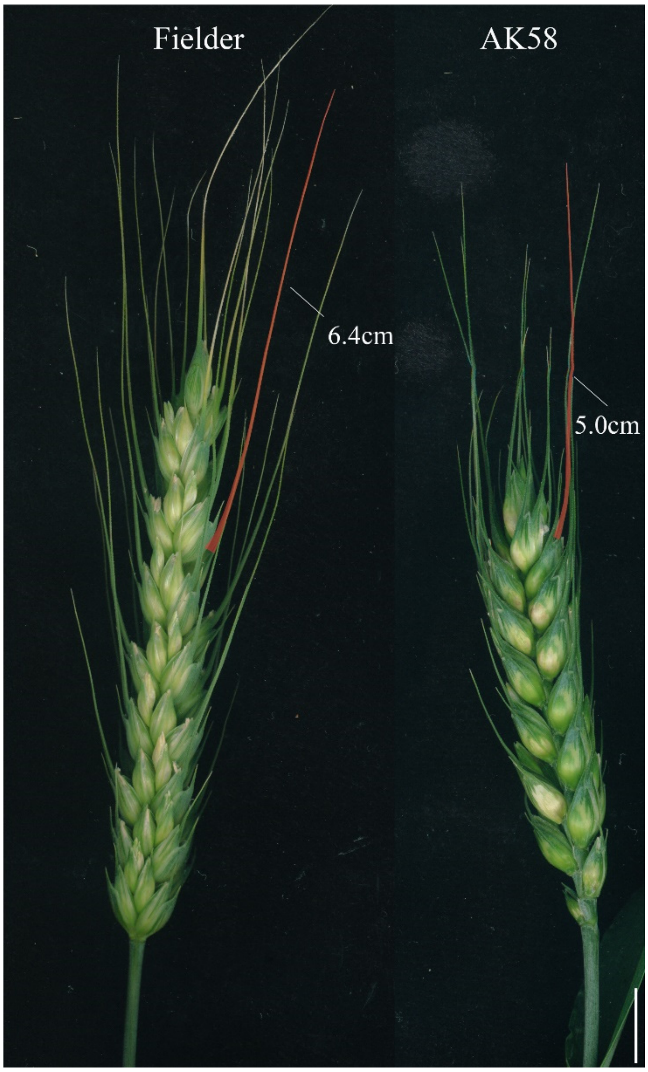

Figure S1. Morphology of awns of awned wheat cultivars Fielder and AK58. Examples of the longest awns are indicated in red. Bar = 1 cm.

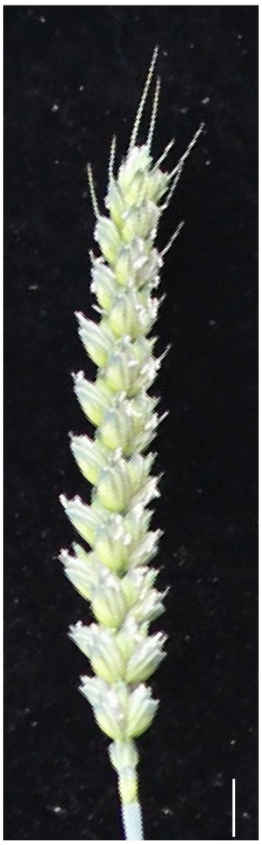

Figure S2. Top awn phenotype of 4045 × ZLWM F_1 plants. Bar = 1 cm.

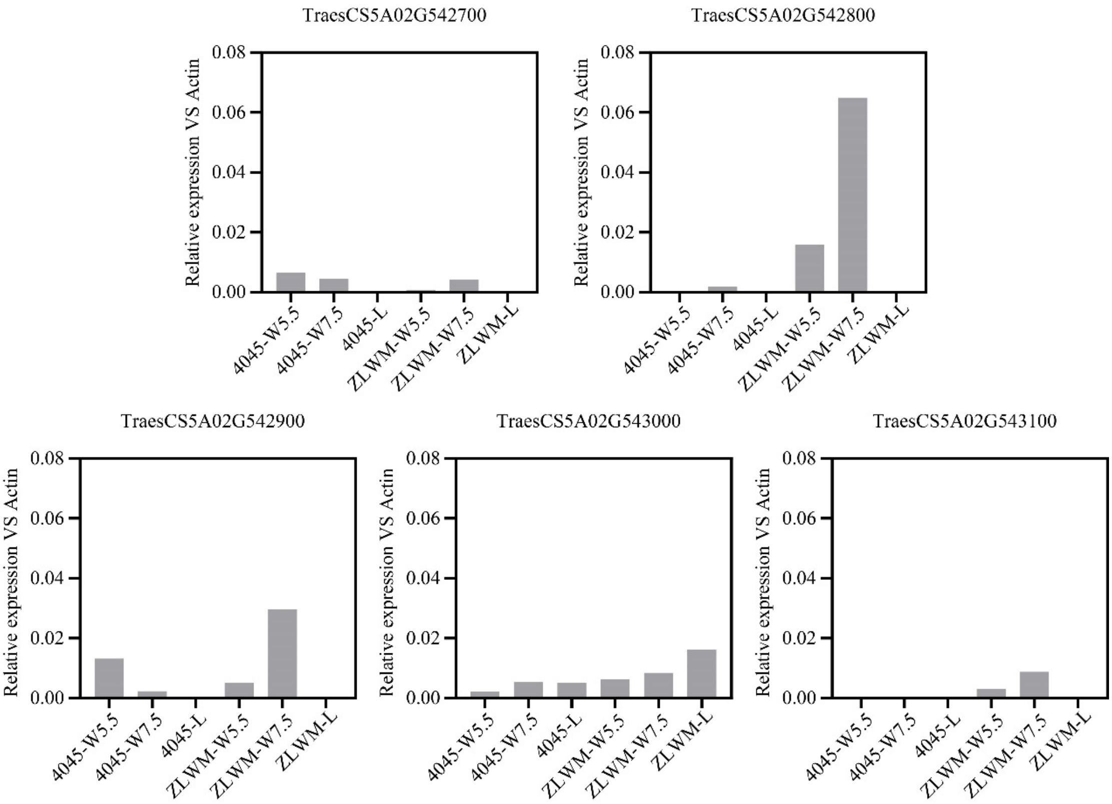

Figure S3. The relative expression versus *TaActin* of the five genes detected by qRT-PCR in young spikes and mature lemma of ZLWM and 4045.

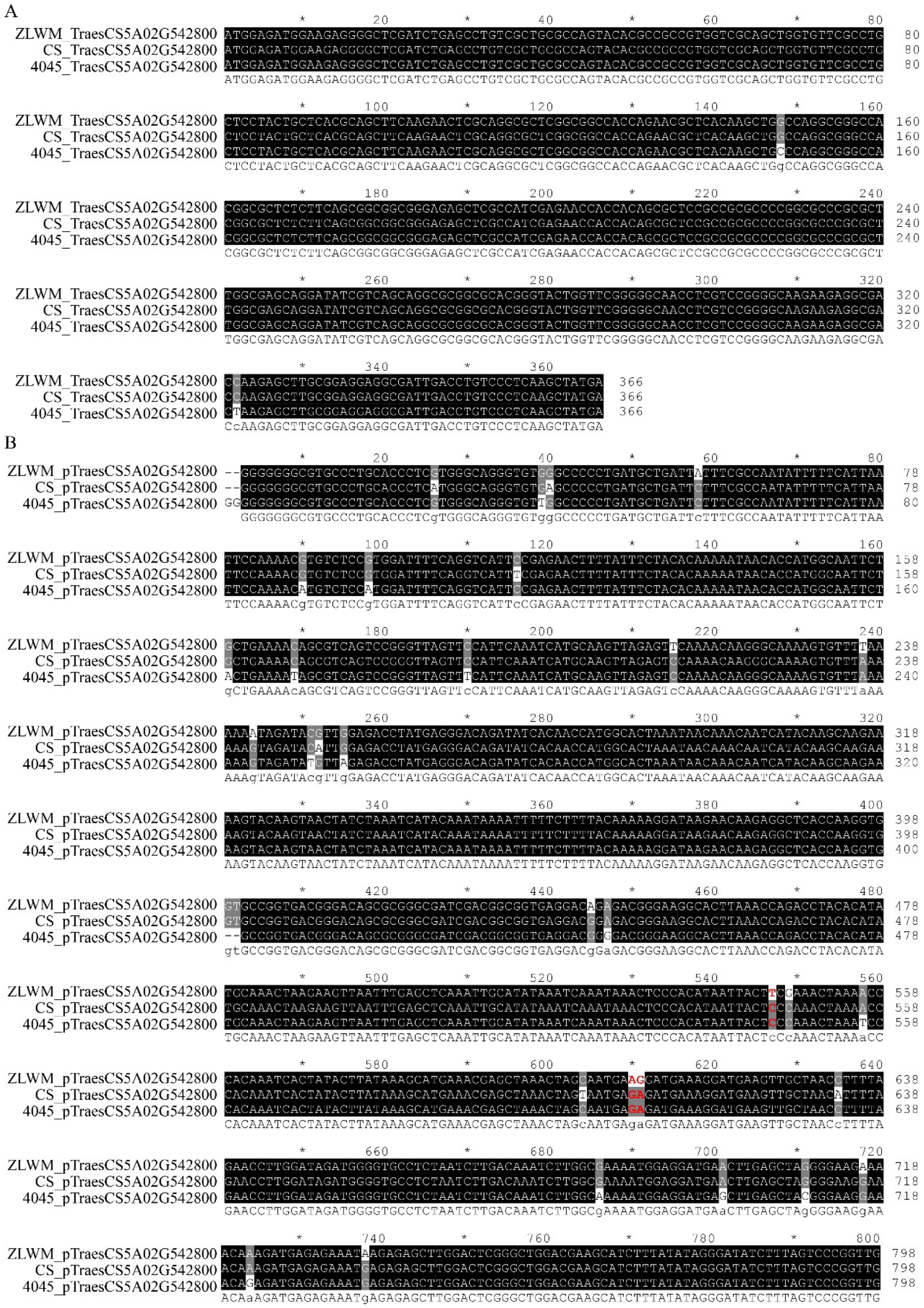
A
ZLWM_TraesCS5A02G542800
CS_TraesCS5A02G542800
4045_TraesCS5A02G542800
B
ZLWM_pTraesCS5A02G542800
CS_pTraesCS5A02G542800
4045_pTraesCS5A02G542800

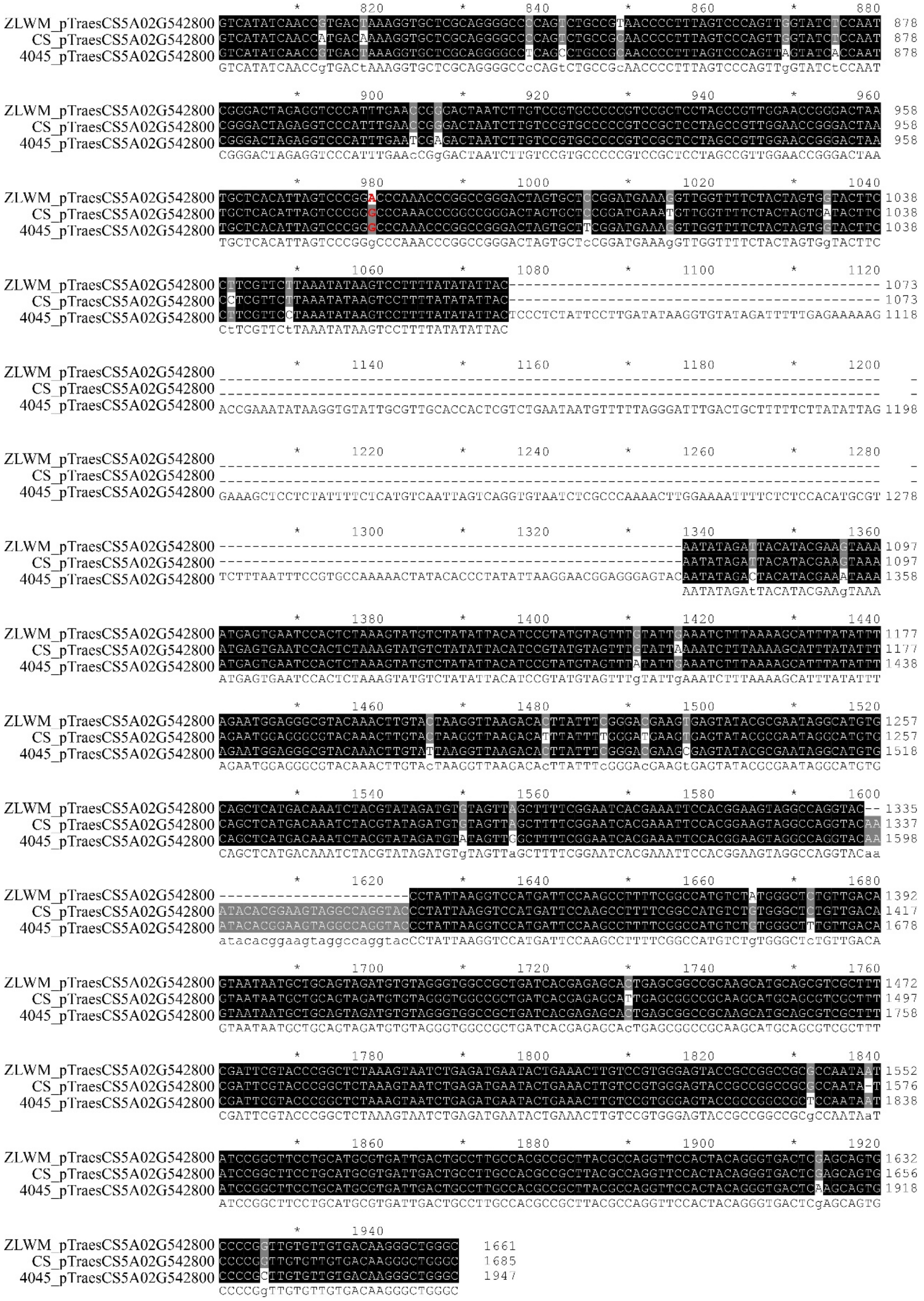

Figure S4. Nucleotide sequence alignments of the coding region **(A)** and promoter region **(B)** of *TraesCS5A02G542800* in ZLWM, CS, and 4045.

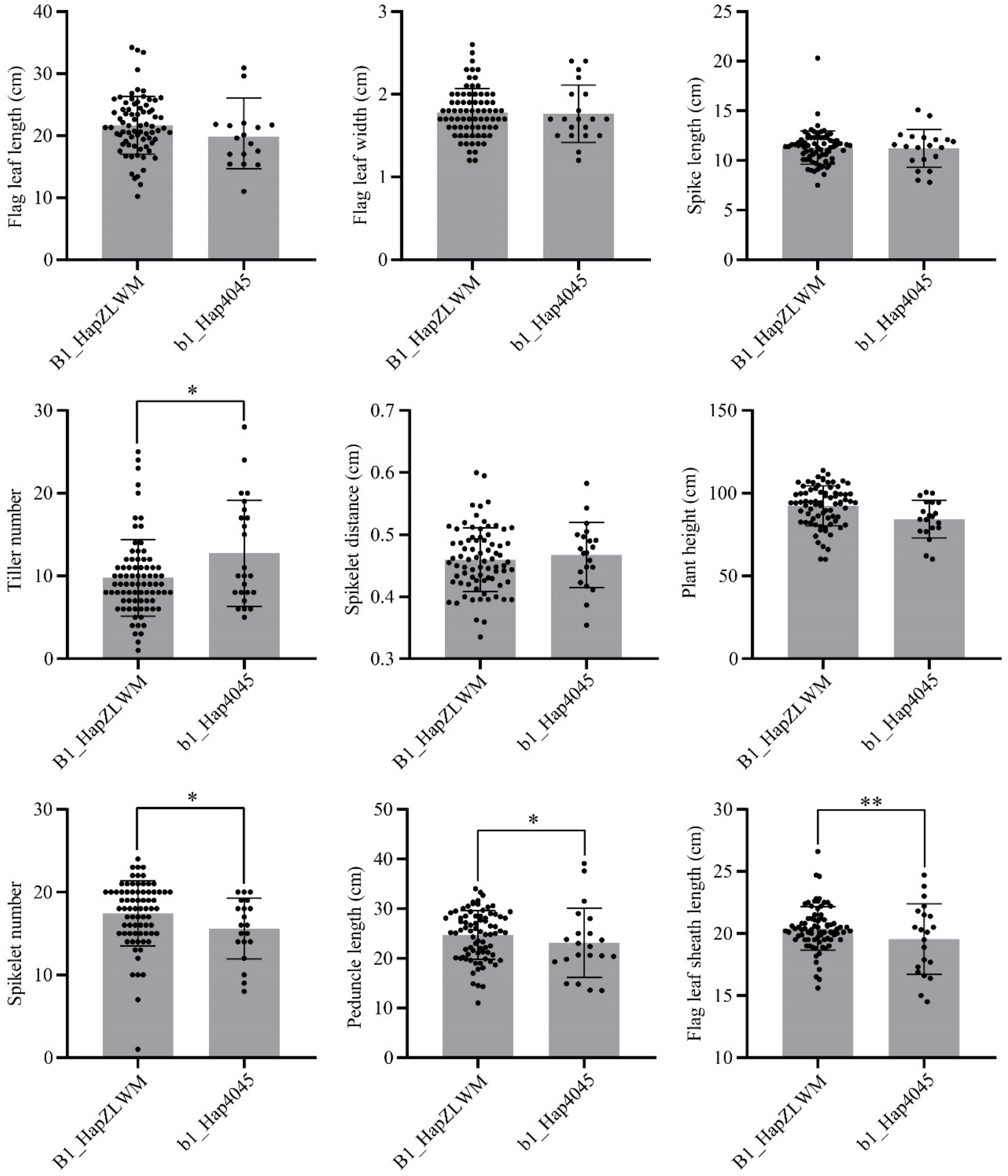

Figure S5. Comparisons of major agronomic traits for haplotypes B1_HapZLWM andb1_Hap4045. n ≥ 74 for B1_HapZLWM, n ⩾ 17 for b1_Hap4045. Each spot indicates a value of the agronomic traits. * indicates $p < 0.05$ by Student's t-test; ** indicates $p < 0.01$ by Student's t-test.

Table S1. Information of KASP markers.

Marker ID	Chr	position (Refseq_V1.1)	Gene	Primer_FAM	Primer_HEX	Primer_Commen
B1_SNP32	5A	697764096	TraesCS5A02G539900	aagcttttctacGcAacGaaC	aagcttttctacGcAacGaaG	acctgatatacggtagcattAagG
B1_SNP51	5A	697764543	TraesCS5A02G539900	ggccctggacatgattcactC	ggccctggacatgattcactT	TgatgctGGTctcctaacCctC
B1_SNP66	5A	698510686	TraesCS5A02G542600	tcgtcttcttcttcctgccG	tcgtcttcttcttcctgccA	cctcttccagaaccagtgcT
B1_SNP74	5A	699320125	\	acggtggatccctgctatcG	acggtggatccctgctatcT	catgcaggaaccgaggtgg
B1_SNP81	5A	699467448	TraesCS5A02G543200	atcaagtcaagccattttgtacttA	atcaagtcaagccattttgtacttC	gcttgtgtaactagctgttggA
B1_SNP21	5A	699467893	TraesCS5A02G543200	gcttcagatgatgatttggagtcG	gcttcagatgatgatttggagtcA	ggtacgacatcgctAtttggT
B1_SNP87	5A	699477761	TraesCS5A02G543300	cagcaatggagggggtgG	cagcaatggagggggtgC	cgacctggacgacatcaaca
B1_SNP95	5A	700351074	TraesCS5A02G544900	tcGacCtgAttgtagagacgA	tcGacCtgAttgtagagacgG	aaaagtatTgcagacttgtaccaG
B1_SNP101	5A	700351698	TraesCS5A02G544900	tgtagttggagtccgtcacG	tgtagttggagtccgtcacC	cgcgatcgagttcctgagA
B1_SNP107	5A	700441208	TraesCS5A02G545600	ccCtgTgcttgtttTgccCG	ccCtgTgcttgtttTgccCA	tgcggAtcAtcaCcattcctgA
B1_SNP27	5A	700441294	TraesCS5A02G545600	atacagaagaaagaatgtggcataG	atacagaagaaagaatgtggcataC	GcACtagctgggcatgAG

Table S2. Information of primers used in this study.

Gene identifier	Primer name	Primer sequence (5'->3')	Purpose
TraesCS5A02G542800	5428F	ATGCGTGATTGACTGCCTTGC	Cloning and sequencing of TraesCS5A02G542800 encoding region
TraesCS5A02G542800	5428R	CCACCCTTCTTGTTCTTCATA	Cloning and sequencing of TraesCS5A02G542800
TraesCS5A02G542800	5428-F1	CCCaagcttATGGAGATGGAAGAGGGGCT	Construct for over-expression
TraesCS5A02G542800	5428-R1	GGggtaccTCATAGCTTGAGGGACAGGT	Construct for over-expression
TraesCS5A02G542800	5428PF1	ATAACATAGGGTCACATAGAAC	Cloning and sequencing of TraesCS5A02G542800 promoter region
TraesCS5A02G542800	5428PF2	ACTCCTCAAACTACGGTCAT	Cloning and sequencing of TraesCS5A02G542800 promoter region
TraesCS5A02G542800	5428T-F1	GGTATACATGTTGATGCGGGTT	Screening for positive transgenic plants
TraesCS5A02G542800	5428T-R1	TCATAGCTTGAGGGACAGGT	Screening for positive transgenic plants
TraesCS5A02G542800	5428T-F2	ATGGAGATGGAAGAGGGGCT	Alternative primer for screening positive transgenic plants
TraesCS5A02G542800	5428T-R2	CGCGCGATAATTTATCCTAGT	Alternative primer for screening positive transgenic plants
TraesCS1A02G274400<br>TraesCS1B02G283900<br>TraesCS1D02G274400	TaActin-F	TGTTGTTCTCAGTGGAGGTTCT	qRT-PCR, used as an endogenous control
TraesCS1A02G274400<br>TraesCS1B02G283900<br>TraesCS1D02G274400	TaActin-R	CTGTATTTCCTTTCAGGTGGTG	qRT-PCR, used as an endogenous control
TraesCS5A02G542700	27RT-F	CGGAACTGGGTCTAATGGGACT	qRT-PCR
TraesCS5A02G542700	27RT-R	CTTGATTGAAATGCTTGGTACTGGAGG	qRT-PCR
TraesCS5A02G542800	28RT-F	GGAGATGGAAGAGGGGCTCGAT	qRT-PCR
TraesCS5A02G542800	28RT-R	TTGAAGCTGCGTGAGCAGTAGG	qRT-PCR
TraesCS5A02G542900	29RT-F	CAGTCCCTCCTGCGAATC	qRT-PCR
TraesCS5A02G542900	29RT-R	AGCATCACAGCCTCAGACC	qRT-PCR
TraesCS5A02G543000	30RT-F	GTGACGGAGGTGCTGGGCATTG	qRT-PCR
TraesCS5A02G543000	30RT-R	AACGGCGGCACGACACTTGG	qRT-PCR
TraesCS5A02G543100	31RT-F	CGGGACGCTGGCAAAGAA	qRT-PCR
TraesCS5A02G543100	31RT-R	CTGGCAGACGATGGTGAGGA	qRT-PCR
